# Functional positioning in robotic lateral unicompartmental knee arthroplasty: a step-by-step technique

**DOI:** 10.1051/sicotj/2025055

**Published:** 2026-02-09

**Authors:** Luca Andriollo, Giovan Giuseppe Mazzella, Filippo Leggieri, Elvire Servien, Cécile Batailler, Sébastien Henri Michel Lustig

**Affiliations:** 1 Orthopaedics Surgery and Sports Medicine Department, FIFA Medical Center of Excellence, Croix-Rousse Hospital, Hospices Civils de Lyon, Lyon North University Hospital 69004 Lyon France; 2 Ortopedia e Traumatologia, Fondazione Poliambulanza Istituto Ospedaliero 25124 Brescia Italy; 3 LIBM-EA 7424, Interuniversity Laboratory of Biology of Mobility, Claude Bernard Lyon 1 University 69100 Lyon France; 4 Univ Lyon, Claude Bernard Lyon 1 University, IFSTTAR, LBMC UMR_T9406 69622 Lyon France

**Keywords:** Functional positioning, Personalized knee arthroplasty, Robotic knee, Unicompartmental knee arthroplasty, UKA

## Abstract

Lateral unicompartmental knee arthroplasty (UKA) represents 1–2% of knee replacement procedures, yet offers distinct advantages including reduced surgical burden, bone stock preservation, and faster functional recovery. However, lateral UKA presents unique technical difficulties due to the surgical complexity of the lateral compartment. Recent advances in image-based robotic systems have demonstrated improved accuracy in implant positioning and promoted more individualized surgical strategies. This article presents a step-by-step surgical technique for lateral UKA using Functional Positioning (FP) principles in combination with an image-based robotic system. The technique ensures precise preoperative planning based on CT imaging, real-time intraoperative kinematic evaluation, and accurate component placement tailored to individual patient anatomy. The key steps of this surgical technique include comprehensive preoperative planning with 3D anatomical modeling, intraoperative kinematic evaluation following osteophyte removal, achieving centered femorotibial contact points throughout the full range of motion with precise lateral laxity gap boundaries, and cartilage mapping to ensure optimal component positioning and avoid overstuffing. FP addresses the characteristic posterior cartilage wear pattern of valgus knees while preserving pre-arthritic coronal alignment and avoiding varus overcorrection. This systematic approach demonstrates reproducible surgical steps that may translate into improved long-term outcomes and implant survivorship for lateral UKA procedures.

## Introduction

Lateral unicompartmental knee arthroplasty (UKA) is performed in only about 1–2% of knee replacement cases [1, 2]. Lateral UKA offers distinct advantages, including reduced surgical invasiveness, preservation of native bone stock and ligaments, improved functional outcomes, higher patient satisfaction, and faster recovery [3].

Nevertheless, lateral UKA presents unique technical challenges compared to medial UKA, largely due to the more complex biomechanics and greater physiological laxity of the lateral compartment, which increase the risk of early failure when implant positioning is suboptimal [4, 5].

Image-based robotic systems have been demonstrated to have better accuracy in implant positioning and postoperative limb alignment [6, 7] and the introduction of robotic assistance has also promoted more individualized surgical strategies [8].

Functional positioning (FP) aims to personalize lateral UKA through precise resurfacing, respecting native anatomy, knee kinematics, and soft tissue balance with the aid of advanced imaging technologies.

This article will describe and illustrate the application of FP to lateral UKA using an image-based robotic system.

## Surgical technique

The surgical technique of functional positioning in lateral UKA is demonstrated in Video 1. Current lateral UKA indications have been previously described in detail by Foissey et al. [9]. The patient is positioned supine on the operating table, with one arm abducted and supported laterally, while the opposite arm rests on the surgical table. A lateral support pad is placed over the mid-thigh, and a distal pad is used to maintain the knee in 90° of flexion during the procedure.

### Step 1: Pre-operative planning

Pre-operative planning involves positioning the fixed-bearing metal-backed unicompartmental components (RESTORIS MCK^®^ partial knee implant system, Stryker^®^, Mahwah, USA), using a dedicated navigation system (Orthomap ASM^®^, Stryker^®^, Mahwah, USA). Planning is based on a preoperative CT scan and the generation of a 3D anatomical model.

At this stage, the implant position is selected to match the patient-specific bone anatomy and restore the pre-arthritic coronal alignment. Intraoperative adjustments are expected.

The aim is to restore the hip-knee-ankle (HKA) angle to between 180° and 185°, a posterior tibial slope ranging from 0° to 12°, a tibial valgus from 0° to 3°, and a ±2 mm change in the joint line (JL) height.

### Step 2: Surgical approach and pin placement

A straight anterolateral skin incision is made, followed by an arthrotomy via a lateral approach. It is essential to perform a cartilage and ligament check of all compartments first to ensure osteoarthritis exclusively affects the lateral compartment.

**Figure 1:**
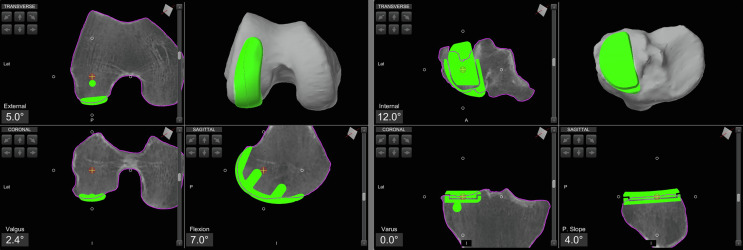
Lateral unicompartmental knee arthroplasty preoperative planning using the Mako^®^ image-based robotic system (Stryker^®^, Mahwah, USA).

Robotic assistance is provided using the Mako^®^ *System* (Stryker^®^, Mahwah, USA). Femoral and tibial pins with optical arrays are placed away from the incision; checkpoints are positioned. The hip center of rotation, bony landmarks, and cartilage thickness are recorded and matched to the pre-operative CT-based model (Figure 1).

### Step 3: Initial knee kinematic evaluation

After bone mapping and matching with the CT imaging, osteophytes from the lateral femoral and tibial condyles and those from the margins of the intercondylar notch are removed. All overhanging osteophytes should be excised prior to assessing ligamentous compliance to avoid interference with the evaluation of the knee kinematics.

After osteophyte removal, kinematic data are acquired, including range of motion (ROM) and lateral soft tissue compliance, through varus stress. To properly balance the gaps throughout the full ROM and collect data from femorotibial tracking, the knee is positioned at different flexion angles, and specific poses are recorded, while applying a varus stress to passively correct the coronal deformity.

### Step 4: Intra-operative planning

During planning refinement, the aim is to achieve centered femorotibial contact points between the components throughout the full ROM, and to achieve 0–2 mm of laxity in the lateral compartment. When determining the laxity curve pattern for each specific pose at various degrees of knee flexion, the goal is to achieve between 0 and 1 mm in extension and higher mm in flexion up to 3–4, to restore the native lateral flexion gap. At this stage, a match should be achieved between the ideal load line of the prosthetic component, visible on the screen, and the anatomical load line of the knee, marked during the kinematic evaluation.

The accuracy of the planning also aims to maintain the neutral sagittal alignment, avoiding fixed flexion or hyperextension.

Determining the correct tibial rotation is challenging, even with the use of CT imaging. The medial edge of the tibial component is first aligned to match the medial cortical edge of the lateral femoral condyle in mid-flexion. Additionally, the position of the femoral and tibial components is verified intraoperatively and prior to bone cuts throughout the entire range of motion using the 3D planning features of the software.

Final fine-tune adjustments to the implant placement are made based on the dimensions of the femoral and tibial components (Table 1).

**Table 1:** Functional positioning protocol guidelines in lateral unicompartmental knee arthroplasty [HKA: hip-knee-ankle angle; JLO: joint line obliquity; CPAK: coronal plane alignment of the knee].

Parameter	Target
Final coronal alignment (HKA)	180°–185°
Final sagittal alignment with gravity only	0° ± 5°
*Femur*	
Varus/valgus	Femoral resurfacing aims to obtain centered contact points between the femoral and tibial implants throughout the full range of motion
Flexion	
Rotation	
*Tibia*	
Varus/valgus	0°–3° valgus
Slope	0°–12°
Rotation	Alignment of the implant’s lateral edge with the lateral cortex axis of the lateral femoral condyle
JLO and height	Preservation of the JLO orientation
	Final joint line height ±2 mm from native
Balancing	0–2 mm of final lateral laxity
	Goal: 0–1 mm in extension more laxity in flexion (up to 3–4 mm) in order to restore the native lateral flexion gap

**Figure 2:**
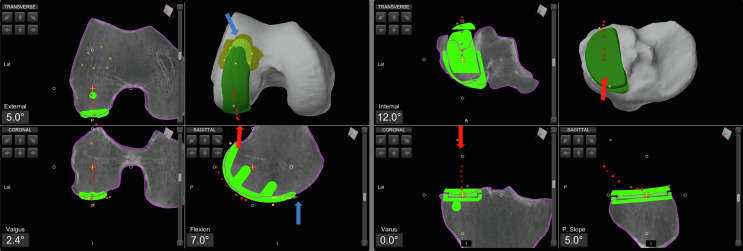
Intraoperative screenshot of lateral unicompartmental knee arthroplasty using Mako^®^ (Stryker^®^, Mahwah, USA), with planning modified according to Functional Positioning principles. The blue arrow indicates the points of femoral cartilage mapping, while the red arrow shows the anatomical load line of the knee, marked during the kinematic evaluation.

Before the end of the planning, the probe is used to map the cartilage alongside the femoral component, to confirm the component has been positioned flush with the cartilage to avoid overstuffing or understuffing. In fact, a prominent anterior tip of the femoral component can impinge on the patella during flexion, potentially leading to disease progression or pain. Cartilage mapping ensures a smooth transition from the femoral component to the anterior surface of the femoral condyle (Figure 2).

### Step 5: Bone preparation and trial component implant

Bone cuts are performed using the arm-assisted saw and burr. Trial components are inserted, beginning with an 8 mm liner, which may be adjusted to optimize varus-valgus stability, considering that a progressive increase in polyethylene thickness will result in a slightly progressive increase in postoperative varus alignment. Laxity and knee kinematic alignment are evaluated during the final knee kinematic evaluation, with immediate computer feedback.

### Step 6: Final implant cementation and closure

The cementation of the final components is performed, with the tibial component cemented first, followed by the femoral component, using progressive compression. Excess cement is removed, and the definitive liner is inserted. After allowing sufficient time for the cement to set, capsular and skin closure is performed.

## Discussion

FP is a three-dimensional alignment framework that utilizes image-based robotic-assisted technology to perform UKA, aiming to restore the native kinematic biomechanics of the lateral compartment of the knee joint in both the coronal and sagittal planes within defined boundaries and to make fine-tune adjustments to the implant positioning based on optical navigation quantitative data of lateral compartment soft tissue laxity patterns. FP also recognizes that varus and valgus morphotypes exhibit distinctly different biomechanical behaviors, necessitating individualized surgical approaches tailored to each knee morphotype [8].

The use of image-based robotic systems enables surgeons to accurately restore the native tibiofemoral contact points of the valgus knee throughout the full range of motion and to preserve the patients’ preoperative laxity gaps with great precision following the intraoperative optical navigation data during stressed balance assessment. FP in lateral UKA enables surgeons to address the characteristic posterior cartilage wear pattern of valgus knees by using robotic guidance to optimize femoral cuts for proper lateral compartment resurfacing, overcoming the anatomical mismatch between standard unicompartmental components designed for anterolateral wear in varus knees. This may translate into long-term greater functional outcomes, higher patient satisfaction rates, and preservation of natural knee biomechanics, which is essential for high-demand activities and long-term implant survivorship [9, 10].

FP boundaries also accommodate the individual lateral compartment laxity pattern and anatomy, representing a resurfacing approach that addresses both the preservation of the pre-arthritic phenotype of the joint and the bone stock. As the most frequent reason for revision following lateral UKA is osteoarthritis progression, FP in lateral UKA has the potential to maintain the pre-arthritic coronal limb alignment and avoid varus overcorrection, thus potentially ensuring long-term survivorship [11]. The use of image-based robotic systems within FP boundaries may enable the surgeon to pursue a resurfacing technique while avoiding undue bone thickness cuts that may prevent tibial failure and preserve bone stock, especially important for osteoporotic bone in valgus morphotypes [12, 13].

Lateral UKA is not common due to the perceived surgical complexity and the lack of consensus-based indications [4, 5, 14]. This study’s detailed technique addressed these challenges by creating groundwork for shared practice guidelines. It also has the potential to equalize outcomes across different surgeon experience levels and establish a methodological foundation for evidence-based evaluation of long-term meaningful benefits in personalized lateral UKA approaches.

This study has several limitations that should be acknowledged. The FP approach described represents a technical framework based on biomechanical principles and surgical experience rather than a comprehensive clinical evaluation with long-term patient outcomes. The learning curve associated with image-based robotic systems may also initially present challenges for surgeons unfamiliar with these technologies, potentially affecting the learning. Furthermore, patient selection criteria for the lateral UKA remain to be fully standardized.

## Conclusions

The FP for lateral UKA provides a systematic framework to achieve personalized implant positioning, preserving native knee kinematics and addressing the unique characteristics of valgus morphotypes. This technique demonstrates reproducible surgical steps to achieve optimal implant positioning tailored to individual patient anatomy that may translate into improved long-term outcomes and implant survivorship.

## Data Availability

The video is available as supplementary material.

## Supplementary material

*Video 1*. Surgical technique. The video is available at the following https://sicotj.edpsciences.org/10.1051/sicotj/2025055/olm.
